# Characterization and Roles of Cherry Valley Duck NLRP3 in Innate Immunity During Avian Pathogenic *Escherichia coli* Infection

**DOI:** 10.3389/fimmu.2018.02300

**Published:** 2018-10-08

**Authors:** Rong Li, Jing Lin, Xiaolan Hou, Shaojie Han, Hongyu Weng, Ting Xu, Ning Li, Tongjie Chai, Liangmeng Wei

**Affiliations:** ^1^Shandong Provincial Key Laboratory of Animal Biotechnology and Disease Control and Prevention, Shandong Provincial Engineering Technology Research Center of Animal Disease Control and Prevention, College of Animal Science and Veterinary Medicine, Sino-German Cooperative Research Centre for Zoonosis of Animal Origin of Shandong Province, Shandong Agricultural University, Tai'an, China; ^2^Collaborative Innovation Centre for the Origin and Control of Emerging Infectious Diseases of Taishan Medical College, Tai'an, China

**Keywords:** Cherry valley duck NLRP3, avian pathogenic *Escherichia coli*, NLRP3-lentiviral vector, innate immunity, cytokine, antibacterial activity

## Abstract

The nucleotide-binding oligomerization domain-like receptor (NLR) pyrin domain containing 3 (NLRP3) is a pattern recognition receptor that is involved in host innate immunity and located in the cytoplasm. In the present study, the full-length cDNA of Cherry Valley duck NLRP3 (duNLRP3) (2,805 bp encode 935 amino acids) was firstly cloned from the spleen of healthy Cherry Valley ducks, and the phylogenetic tree indicated that the duNLRP3 has the closest relationship with *Anas platyrhynchos* in the bird branch. According to quantitative real-time PCR analysis, the duNLRP3 mRNA has a broad expression spectrum in healthy Cherry Valley duck tissues, and the highest expression is in the pancreas. There was significant up-regulation of duNLRP3 mRNA expression in the liver and down-regulation in the spleen after infection with avian pathogenic *Escherichia coli* (APEC) *O1K1*, especially at 3 days after the infection. Ducks hatched from NLRP3-lentiviral vector-injected eggs had significantly higher duNLRP3 mRNA expression in the liver, spleen, brain, and cecum, which are tissues usually with lower background expression. The mRNA expression levels of inflammatory cytokines IL-1β, IL-18, and TNF-α significantly increased after the APEC infection in those tissues. The bacterial content in the liver and spleen decreased significantly compared with the NC-lentiviral vector-injected ducks. In addition, in the duck embryo fibroblasts, both of the overexpression and knockdown of duNLRP3 can trigger the innate immune response during the *E. coli* infection. Specifically, overexpression induced antibacterial activation, and knockdown reduced the antibacterial activity of the host cells. The IL-1β, IL-18, and TNF-α mRNA expressions showed up-regulation or down-regulation. The results demonstrate that duNLRP3 has a certain antibacterial activity during *E. coli* infection. These findings also contribute to better understanding the importance of duNLRP3 in regulating the inflammatory response and the innate immune system of ducks.

## Introduction

Pattern recognition receptors (PRRs) are a series of innate immunity receptors that are encoded by the germline and expressed in various kinds of cells for the detection of various microbial components (1, 2). The innate immune response is activated when pathogen- or danger-associated molecular patterns in the cells or the cytoplasm are recognized by the PRRs, and downstream antibacterial or antiviral proteins are induced (3, 4). Nucleotide binding oligomerization domain (NOD)-like receptor (NLR) proteins are receptors in the family of PRRs that are found in the cytosol and detect a wide range of pathogens (bacteria, fungi, and viruses), tissue damage, and other cellular stresses (5).

The NLRs constitute three major domains: a central nucleotide-binding and oligomerization (NACHT) domain, a domain with a variable number of C-terminal leucine-rich repeats (LRRs), and N-terminal caspase recruitment (CARD) or pyrin (PYD) domains (5). The LRRs are believed to be involved in ligand sensing and auto-regulation, and central NACHT is responsible for dNTPase activity and oligomerization, which is a key factor in triggering inflammasome formation. The CARD or PYD domains plays a major role in mediating homotypic protein-protein interactions for downstream signaling (5, 6). At present, the most extensively studied NLRs are NOD1, NOD2, and NLR pyrin domain containing 3 (NLRP3, also known as cryopyrin and NALP3). NLRP3 has PYD domains in its N-terminal and belongs to the NLRP subfamily. Different from NOD1 and NOD2, another important function of NLRP3 is the formation of the inflammasome. The NLRP3 inflammasome is the best-characterized inflammasome and is a multi-protein complex composed of the cytoplasmic innate receptor NLRP3, the adaptor apoptosis-associated speck-like protein containing CARD (ASC), and the effector caspase-1 (7, 8). There are numerous factors that could activate the NLRP3 inflammasome, including sterile and pathogen factors, such as ATP, alum, ultraviolet radiation, and toxins, muramyl dipeptide, RNA, and DNA from bacterial, viral, and fungal pathogens (9). When NLRP3 is activated, the oligomerization of NLRP3 results in clustering of the PYD domain and presents a homotypic interaction with PYD- and CARD-containing adapter ASC. The CARD domain of ASC could in turn recruit the CARD of procaspase-1. Procaspase-1clusters automatically cleave and form the active caspase-1 p10/p20 tetramer and then induce the production of activated IL-1β and IL-18 (10). The functions of inducing the production of IL-1β and IL-18 make the NLRP3 inflammasome play an important role in the inflammatory and innate immune response in vertebrates.

Avian Pathogenic *Escherichia coli* (*E. coli*, APEC) is an important extracellular pathogen that can cause the severe septicemia, perihepatitis, pericarditis, and airsacculitis. It has high morbidity and mortality in ducks and can infect them at various ages (11, 12). Previous studies have shown that *E. coli* O157:H7 stimulates mice and human cells to produce NLRP3 inflammasome-dependent chemokine and proinflammatory mediators (13, 14). These studies indicate that NLRP3 inflammasome activation is closely associated with *E. coli* interaction. Ducks are the main commercial waterfowl species in China, and their intensive farming has brought great profits (15). But over the past years, outbreaks of many diseases such as colibacillosis have brought about huge economic losses in the duck industry.

Research about PRRs in mammals is growing, but there are relatively few reports about waterfowl. Furthermore, the types and functions of PRRs are not the same among different species (16–18). Therefore, we utilized *in vivo* and *in vitro* models in Cherry Valley duck and duck embryo fibroblasts (DEFs) to further the understanding about the innate immune function of duck NLRP3 (duNLRP3) during APEC infection. DuNLRP3 was cloned from healthy Cherry Valley duck spleen, and the activity of NLRP3 and innate immune response after the APEC infection were studied based on the exogenous overexpression (NLRP3-lentiviral vector) and the knockdown of endogenous duNLRP3 (Si-NLRP3). This is the first report on the importance of duNLRP3 in regulating the inflammatory response and influencing the progression of APEC infection.

## Materials and methods

### Eggs, cells and bacteria strain

Cherry Valley duck eggs were purchased from a farm in Tai'an, Shandong China. They were incubated at 37.5°C with a relative humidity of 55–65% and rocked at a 90° angle at 2 h intervals for microinjection at 3 days.

DEFs were derived from 12-day-old Cherry Valley duck embryo and cultured in Dulbecco's modified Eagle medium (DMEM) (Gibco, Grand Island, NY, USA) supplemented with 10% fetal bovine serum (Transgen, Beijing, China). The cells were then incubated at 37°C in 5% (v/v) CO_2._

The APEC *O1:K1* strain was isolated and stored by the Environmental Microbiology Laboratory at Shandong Agricultural University. A single colony was selected from Luria-Bertani (LB) agar and inoculated in LB broth at 37°C overnight. The bacterial broth was then serially diluted by 10-fold and plated on eosin methylene blue nutrient agar to calculate the bacterial content to 10^4^ CFU/mL.

### Cloning, characterization, and phylogenetic analysis of duNLRP3

To study the function of duNLRP3, full-length CDs of duNLRP3 were obtained by one set of specific polymerase chain reaction (PCR) primer duNLRP3 F/R (Table 1) from healthy Cherry Valley ducks' spleen and the full-length cDNA of duNLRP3 was sequenced. The protein number of each species of NLRP3 is shown in Table 2. The structure of the amino acid sequences of duNLRP3 was predicted by the SMART tool. Multiple amino acid sequence alignments were performed using ClustalW2 and edited with the online tool Boxshade. The phylogenetic analysis was generated using MEGA5.1 software, and the tree was constructed by the neighbor-joining method with bootstrapping over 1,000 replicates.

**Table 1:** Primers used in this study.

**Primer name**	**Sequence(5′-3′)**	**Purpose**
duNLRP3 F	ATGGCGGGGGAAGGGAGTGC	Gene cloning
duNLRP3 R	TCAGCAGTGGTTTCTGTTGC	
qd NLRP3 F	ACAGCTTCACACACCTGCAC	qRT-PCR
qd NLRP3 R	GTGAAATTCTGCACCCGATT	
qd IL-1β F	TCATCTTCTACCGCCTGGAC	qRT-PCR
qd IL-1β R	GTAGGTGGCGATGTTGACCT	
qd IL-18 F	CTGATGACGATGAGCTGGAA	qRT-PCR
qd IL-18 R	CAAAAGCTGCCATGTTCAGA	
qd TNF-α F	ACCCCGTTACAGTTCAGACG	qRT-PCR
qd TNF-α R	CTGGTTACAGGAAGGGCAAC	
qd β-actin F	GGTATCGGCAGCAGTCTTA	qRT-PCR
qd β-actin R	TTCACAGAGGCGAGTAACTT	

**Table 2:** Reference species information.

**Species**	**GenBank accession numbers**
*Ailuropoda melanoleuca*	XP_002930408.2
*Canis lupus*	XP_848377.2
*Equus asinus*	XP_014682199.1
*Equus caballus*	XP_014586320.1
*Vicugna pacos*	XP_006211980.1
*Sus scrofa*	NP_001243699.1
*Ovis aries*	XP_012033754.1
*Pantholops hodgsonii*	XP_005977777.1
*Gorilla*	XP_004028766.1
*Homo*	NP_004886.3
*Papio anubis*	XP_003893702.1
*Macaca fascicularis*	XP_005539633.1
*Macaca mulatta*	NP_001107823.1
*Macaca nemestrina*	XP_011727866.1
*Loxodonta africana*	XP_003421233.1
*Mus musculus*	NP_665826.1
*Rattus norvegicus*	NP_001178571.1
*Oryctolagus cuniculus*	XP_002723203.1
*Gallus*	XP_001233262.3

### Microinjection of the lentiviral vector

The negative control (NC)- and NLRP3-lentiviral suspensions were customized from GeneChem Biotechnology Co. Ltd., (Shanghai, China), and the pGCL-eGFP and flag tags were included. Before infection, lentiviral stock was diluted to 10^8^ uTU/mL with DMEM. The upper surface of 3-day-old Cherry Valley duck eggs was then sprayed with 75% ethanol for sterilization. Freshly laid eggs were microinjected with 8 μL (10^8^ TU/mL) of lentiviral vector by yolk sac injection (19). The embryo was marked under a fiber optic light source, and a small hole was made in the shell using a dental drill and tweezers. After the injection, the hole was sealed with paraffin, and the eggs were incubated until hatching. NC-lentiviral injected eggs, hatched embryos, and birds were used as negative controls.

### *In vivo* experimental procedure

All animals used in this study were handled in strict accordance with the guidelines of the Shandong Agricultural University Animal Care and Use Committee. The approval number was SDAU-2016-001.

To examine the tissue distribution of duNLRP3 and whether it is involved in the innate immune response caused by the *E. coli*, 2-week old ducks were infected neck subcutaneously with 0.3 mL of APEC *O1K1* (10^4^ CFU/mL) per duck. The control group was inoculated with the same volume of 0.65% normal saline (20). Three ducks with significant clinical symptoms (listlessness, anorexia, and diarrhea) in each group were killed at 1, 2, and 3 days post-infection (dpi), and the liver, spleen, and brain were immediately preserved in liquid nitrogen for duNLRP3 detection by quantitative real-time PCR (qRT-PCR). In addition, three healthy ducks were selected for the detection of duNLRP3 tissue distribution before the infection. Tissues were collected and analyzed by qRT-PCR from the heart, liver, spleen, lung, kidney, brain, cerebellum, trachea, esophagus, proventriculus, gizzard, duodenum, jejunum, ileum, cecum, rectum, bursa of Fabricius, thymus, pancreas, muscle, and skin.

After hatching, the lentiviral vector-infected ducks were fed normally for 2 weeks, and then three ducks each were randomly selected from both the NC- and NLRP3-lentiviral vector injected groups. And the 21 kinds of tissues as described above were collected for duNLRP3 mRNA and protein expression level detection. The ducks were then divided into two groups: Group I: NC-*E. coli* (0.3 mL 10^4^ CFU/mL *E. coli* per duck subcutaneously via the neck); Group II: NLRP3-*E. coli* (0.3 mL 10^4^ CFU/mL *E. coli* per duck subcutaneously via the neck). At 1, 2, and 3 dpi, three ducks with significant clinical symptoms were killed. The liver, spleen, and brain were immediately preserved in liquid nitrogen for duNLRP3 and inflammatory cytokine detection. The clinical symptoms of the remaining ducks in the infected group were observed until 14 dpi before they were euthanized.

### Si-RNA

Negative control Si-RNA (pSi-NC) and three Si-NLRP3s (pSi-NLRP3-1, pSi-NLRP3-2, and pSi-NLRP3-3) were synthesized by GenePharma (Shanghai, China). Next, 1 μg of pSi-NC or pSi-NLRP3s was transfected to DEFs on 6-well plates with TransIL-LT1 Transfection Reagent (Mirusbio, CA, USA). After 36 h post-transfection (hpt), RNA was extracted from the cell samples. The silencing efficiency of Si-NLRP3s was controlled by pSi-NC and analyzed by qRT-PCR. The sequences of Si-RNA are shown in Table 3.

**Table 3:** The sequences of pSi-RNA.

**pSiRNA**	**positions**	**Sense sequence (5′-3′)**	**Antisense sequence (5′-3′)**
pSi-NC		UUCUCCGAACGUGUCACGUTT	ACGUGACACGUUAGAATT
pSi-NLRP3-1	564	CCACGCUUGUUAACUGCAUTT	AUGCAGUUAACAAGCGUGGTT
pSi-NLRP3-2	911	CCUGAUGAAGAUGGGCAAATT	UUUGCCCAUCUUCAUCAGGTT
pSi-NLRP3-3	1243	GGAAGCAAAGCCACUGGAATT	UUCCAGUGGCUUUGCUUCCTT

### *In vitro* experimental procedure of DEFS

DEFs were plated in 6-well plates 12 h prior to transfection to examine whether duNLRP3 is involved in the innate immune response caused by *E. coli in vitro*. They were then infected with 20 μL of 10^8^ TU/mL NC- or NLRP3-lentiviral vector (MOI = 10) for 72 h or transfected with 1 μg of pSi-NLRP3 or pSi-NC for 36 h. Next, the cells were infected with 10^4^ CFU *E. coli* for 3 h and washed three times with PBS containing gentamicin (100 μg/ml) to kill the extracellular *E. coli*.

The cells were cultured for 3 h in DMEM containing gentamicin and then lysed for 20 min in 500 μL of PBS containing 1% (v/v) Triton X-100. Finally, the cell samples were plated on nutrient agar to count the intracellular bacteria. Parallel cell samples were also harvested for RNA extraction. The lentiviral vector-infected or Si-RNA-transfected cells that were uninfected with *E. coli* were also harvested for bacterial counting and RNA extraction as the control group.

### QRT-PCR

Total RNA of the samples was extracted and reverse transcribed according to the manufacturer's instructions. Table 1 shows the qRT-PCR primers of duNLRP3, inflammatory cytokines IL-1β, IL-18, and TNF-α, and endogenous gene β-actin. QRT-PCR was conducted with ChamQTM SYBR® qPCR Master Mix (Vazyme, Nanjing, China) and the 7500 Fast Real-Time PCR System (Applied Bio-systems, CA, USA). The PCR reactions were done in a 20-μL volume with the following conditions: 1 cycle at 95°C for 5 min, followed by 40 cycles at 95°C for 10 s and at 60°C for 34 s. The dissociation curve was then analyzed. Each sample was analyzed in triplicate.

### Western blot analysis

Cells were washed twice with cold PBS and lysed in RIPA buffer supplemented with a protease inhibitor cocktail (Beyotime). Before the SDS–PAGE, protein samples were mixed with 5×SDS loading buffer, boiled for 10 min, and transferred to PVDF membranes. After blocking with 5% skim milk for 2 h at room temperature, probing was done with primary antibodies (Anti-Flag Tag Mouse Monoclonal Antibody, Abbkine, California, USA), followed by incubation overnight at 4°C. The secondary antibody was then added for 2 h at room temperature (HRP-labeled Goat Anti-Mouse IgG (H + L), Beyotime Institute of Biotechnology, China). A Western ECL Substrate kit was used to obtain protein images with ChemiDoc XRS (Bio-Rad, Marnes-la-Coquette, France).

### Calculations and statistical analysis

The relative expression levels of the tested genes were calculated using the 2^−Δ*Ct*^ and 2^−ΔΔ*Ct*^ method (21). The duck β-actin gene was used as the endogenous control. All data are represented as the means ± SD, and statistical analyses were performed using Graph Pad Prism 5 software (Graph Pad Software Inc., San Diego, CA, United States). The differences were evaluated by student's *t*-test and ANOVA test with the SPSS software (SPSS Inc., Chicago, IL, United States). Statistical significance was set at *P* < 0.05, and *P* < 0.01 indicated high significance.

## Results

### Characterization of duNLRP3

**Figure 1:**
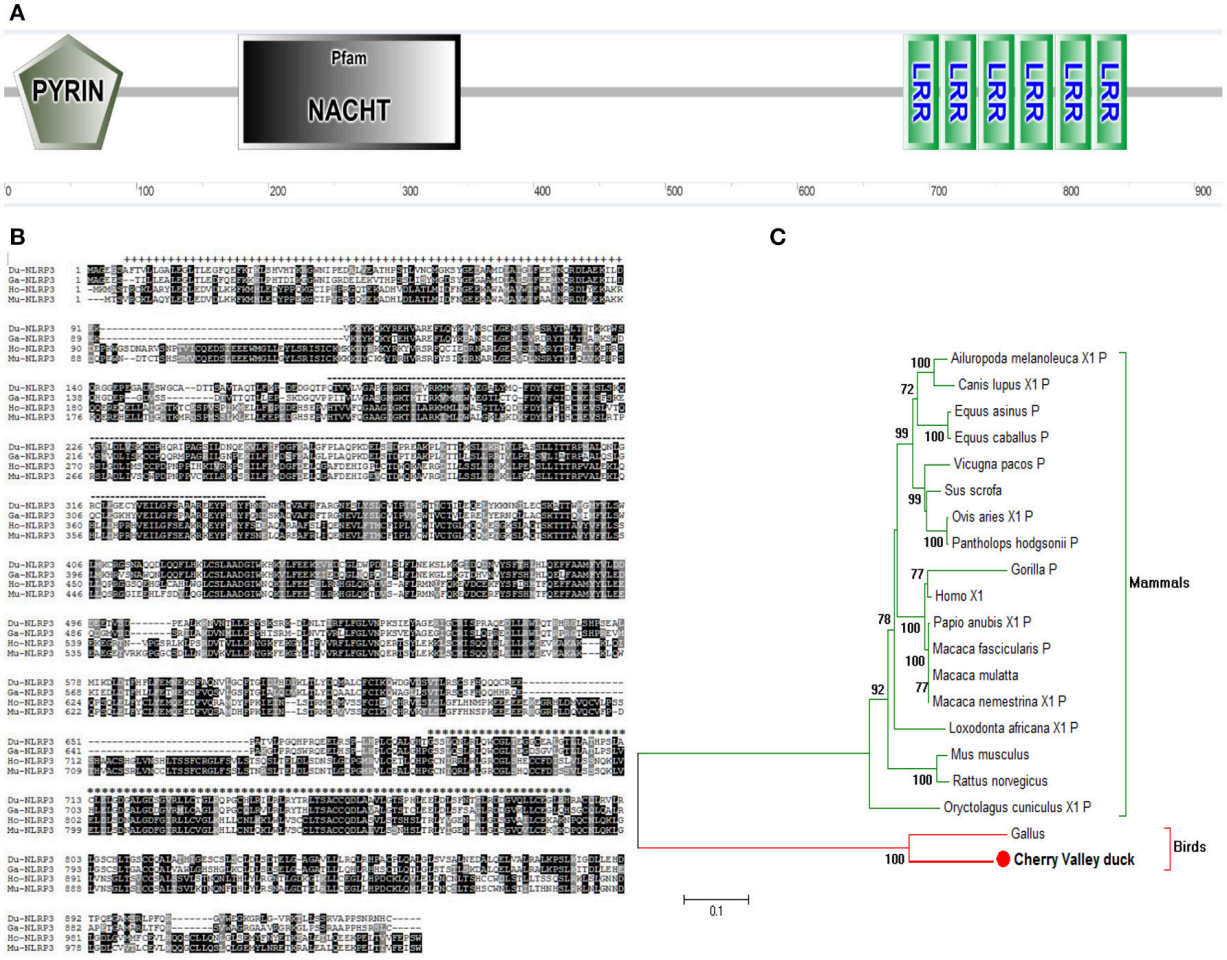
Characterization of duNLRP3. **(A)** Protein motifs of duNLRP3 were analyzed using SMART. **(B)** Multiple alignment of duNLRP3. Alignment was performed using the Clustal X program and edited with Boxshade. The NLRP3 sequences are shown for Cherry Valley duck (Du), *Gallus* (Ga), *Homo* (Ho), and *Mus musculus* (Mu). The amino acid sequences used are shown in Table 2. Black shading indicates amino acid identity, gray shading indicates similarity (50% threshold), + + + above the sequence represents the PYD domains, the black dotted line represents the NACHT domain, and *** the sequence represents the six LRR domains. **(C)** A phylogenic tree based on NLRP3 between Cherry Valley duck and other species' amino acid sequences. The neighbor-joining tree was generated using MEGA 5.0, and a 1000-replicate bootstrap analysis was performed. The scale bar indicates 0.1. GenBank accession numbers are shown in Table 2.

After the cloning, the complete open reading frame of duNLRP3 was obtained. It has a length of 2,805 bp and encodes 934 amino acids. The sequence was submitted to GenBank (MH373356). The secondary structures of the amino acid sequence were predicated by the SMART program, and the results indicated that duNLRP3 contained three characteristic domains of NLRs: an N-terminal PYD domain (7–90aa), a central NACHT domain (177–345aa), and C-terminal six LRR domains (680–707, 708–735, 734–764, 765–793, 795–822, and 823–849aa) (Figures 1A,B). The phylogenetic tree was constructed with full-length NLRP3 protein, and two major branches were observed (mammals and birds). DuNLRP3 was branched with birds, and showed higher evolutionary relationship than with mammals (Figure 1C). These results show that the sequence of NLRP3 in Cherry Valley ducks is relatively conservative and has the highest homology with birds.

### Expression of duNLRP3 *in vivo*

**Figure 2:**
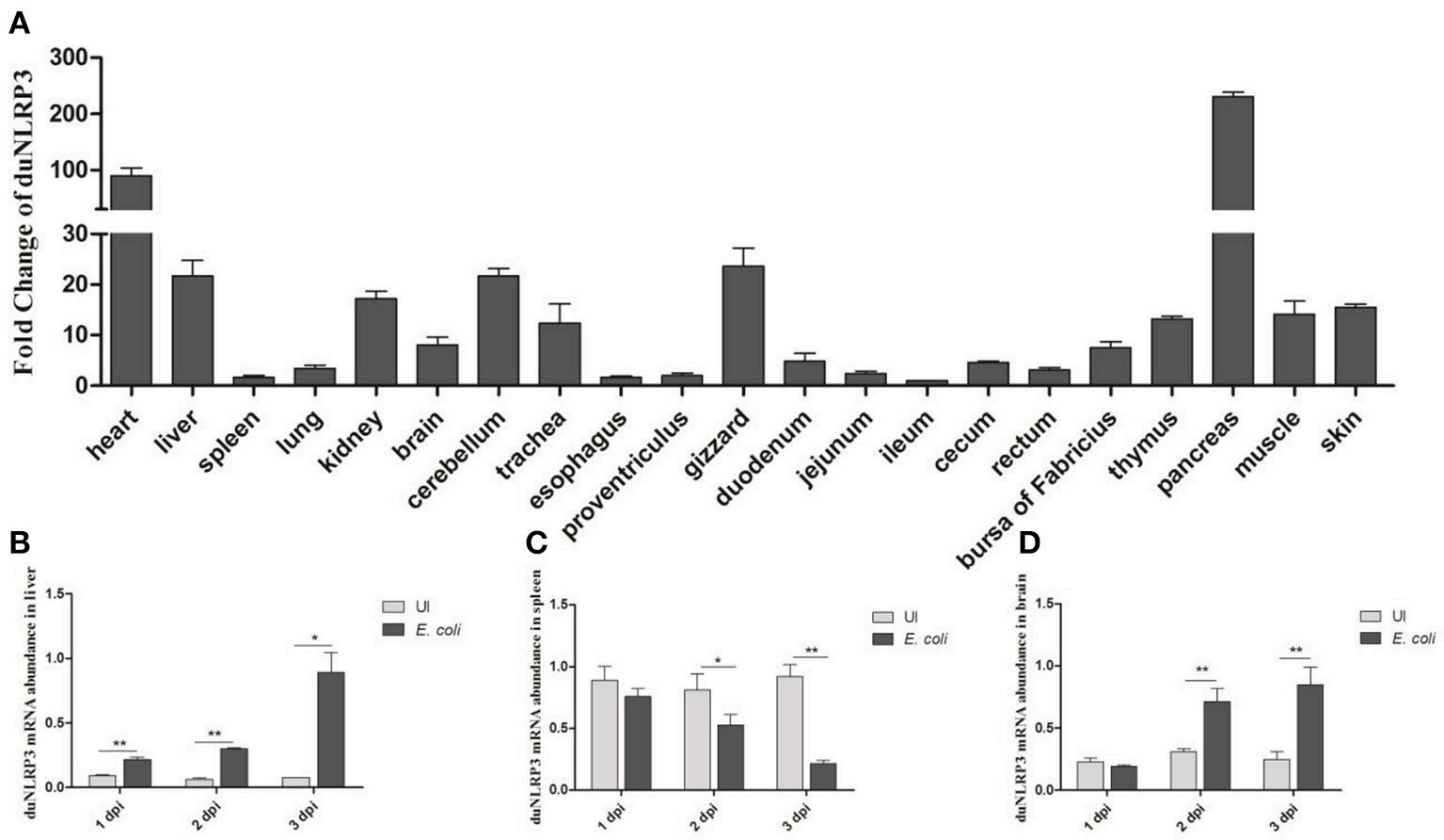
Expression profiles of NLRP3 in Cherry Valley duck. **(A)** Expression of duNLRP3 in tissues of healthy Cherry Valley duck, the ileum was chosen as the standard tissue. Expression of duNLRP3 in the **(B)** liver, **(C)** spleen, and **(D)** brain of Cherry Valley ducks from *E. coli* uninfected group (UI) and *E. coli* infected group (*E. coli*), and these mRNA fold changes were calculated using the *E. coli*-infected ducks vs. control group ducks at the same time point. The relative expressions of duNLRP3 were normalized by β-actin with the 2^−ΔΔ*Ct*^ **(A)** and 2^−Δ*Ct*^ **(B–D)** method. Means ± *SD* (*n* = 3) from three independent repetitions are presented. The student's t test was performed to evaluate the differences. *Significant difference (*P* < 0.05); **highly significant difference (*P* < 0.01); dpi, days post-infection.

To analyse the expression levels of duNLRP3 mRNA in tissues of healthy Cherry Valley ducks, three healthy ducks were randomly selected, and tissues were collected. The ileum was chosen as the standard tissue. As shown in Figure 2A, the highest mRNA expression level of duNLRP3 was observed in the pancreas, followed by the heart, gizzard, and cerebellum. And the mRNA expression level in ileum, spleen, proventriculus, and cecum is lower. The wide expression of duNLRP3 indicates that it might be extensively involved in the host immune response of healthy Cherry Valley ducks. In addition, after the *E. coli* infection, the expression of duNLRP3 displayed a significant up-regulation in the liver at 1–3 dpi, and the fold increases rose with time, reaching a peak at 3 dpi (Figure 2B, *P* < 0.05). There was significant down-regulation in the spleen at 2–3 dpi, and the most significant decrease was observed at 3 dpi (Figure 2C, *P* < 0.01). The expression of duNLRP3 displayed no significant difference at 1 dpi in the brain but was significantly up-regulated at 2 and 3 dpi (Figure 2D, *P* < 0.01). These results suggest that duNLRP3 may be involved in the host innate immunity caused by *E. coli* infection.

### Antibacterial activity of duNLRP3 in the innate immune response *in vivo*

**Figure 3:**
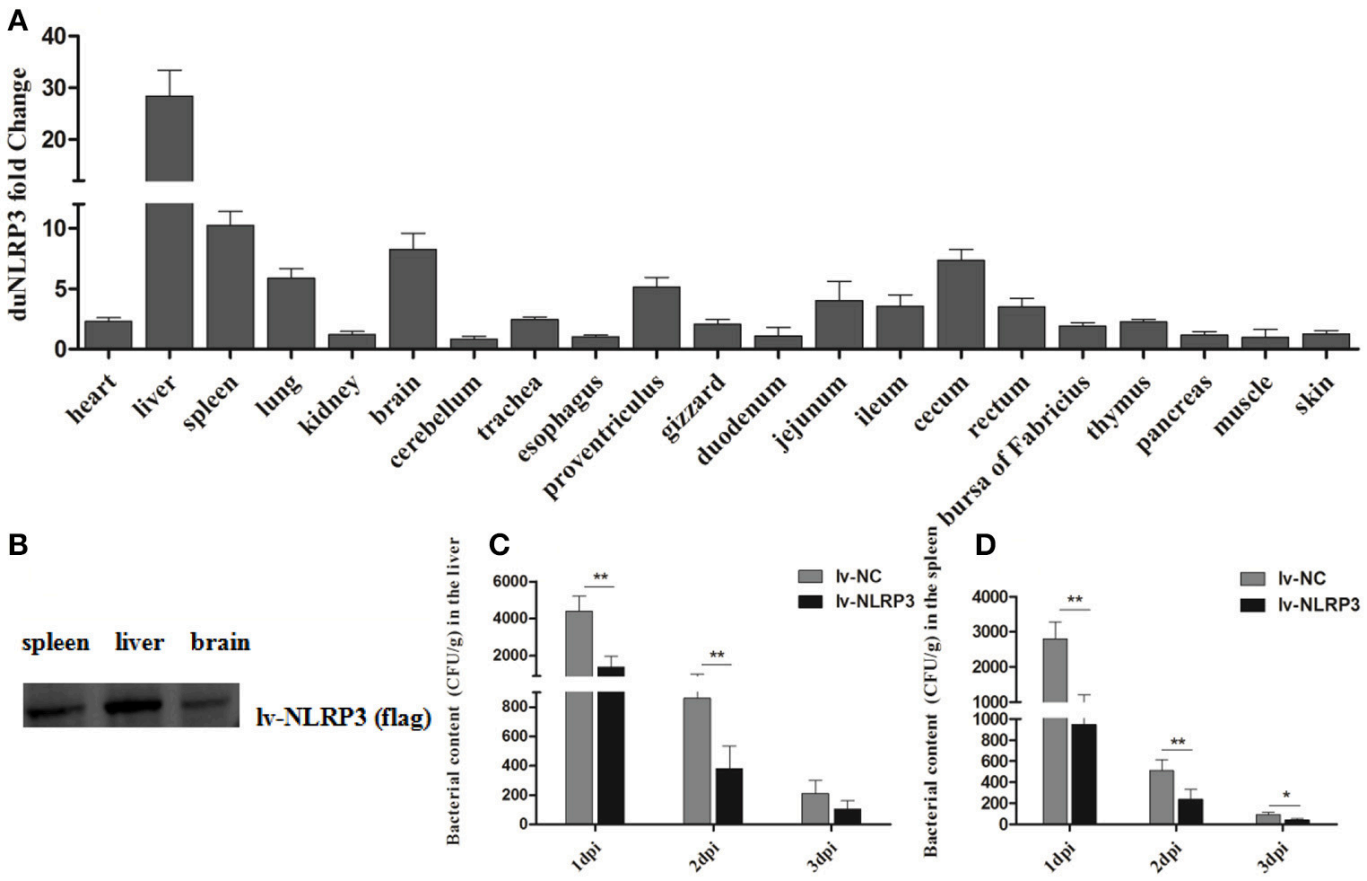
DuNLRP3 reduces the bacteria growth *in vivo***. (A)** Expression of duNLRP3 in tissues of NLRP3-lentiviral vector infected ducks. The duNLRP3 mRNA fold change was calculated using the NLRP3-lentiviral vector-infected ducks vs. NC-lentiviral vector infected ducks at the same time point. The data were analyzed by 2^−ΔΔ*Ct*^ method. **(B)** DuNLRP3 protein expression level in spleen, liver, and brain of NLRP3-lentiviral vector infected ducks. Bacterial content in the lentiviral vector infected ducks' **(C)** liver and **(D)** spleen. All data were expressed as the means ± SD (*n* = 3), and the student's *t* test was performed to evaluate the differences. *Significant difference (*P* < 0.05); **highly significant difference (*P* < 0.01); dpi, days post-infection.

To evaluate the role of duNLRP3 in the *E. coli*-induced innate immune response *in vivo*, ducks hatched from the lentiviral vector injected eggs were infected with *E. coli*. After infection, the NC-*E. coli* group ducks showed the listlessness, anorexia, and diarrhea from 2 dpi and died from the 4 dpi, but NLRP3-*E. coli* group ducks exhibited these typical clinical symptoms from 3 dpi, died from the 6 dpi. At necropsy, the diseased ducks from these two groups both showed serious yellowish–white fibrinous exudate attachment at the heart and liver, as well as the peritoneal fibrosis adhesions. As shown in Figure 3A, the duNLRP3 has a varying degree of overexpression in these tissues. In addition, we also tested the protein expression level of duNLRP3 at these three tissues (Figure 3B). Accordingly, the liver, spleen, and brain were selected for further study. After the *E. coli* infection, the bacterial contents in the liver and spleen were counted. The results show that the bacterial contents in the NLRP3-lentiviral vector-injected group is much lower than the NC-lentiviral vector-injected group at 1–2 dpi (Figures 3C,D, *P* < 0.01). As the main target organ of *E. coli*, the liver's bacterial content was higher than that of the spleen (Figures 3C,D).

**Figure 4:**
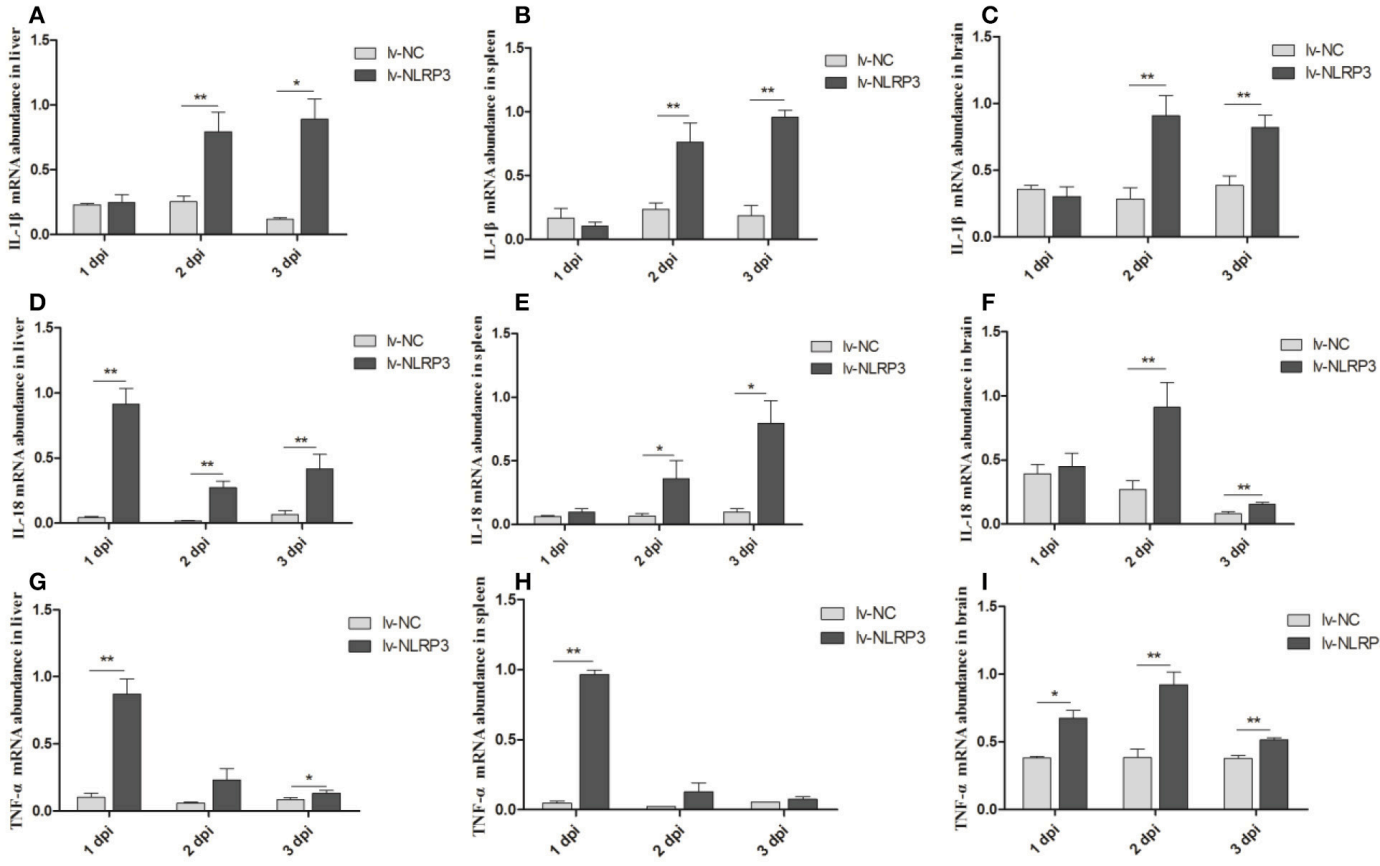
Expression profiles of inflammatory cytokines in lentiviral vector-APEC infected ducks tissues. The mRNA expression abundance of IL-1β in liver **(A)**, spleen **(B)**, brain **(C)**, IL-18 in liver **(D)**, spleen **(E)**, brain **(F)**, and TNF-α in liver **(G)**, spleen **(H)**, brain **(I)** from the NC-lentiviral vector and NLRP3-lentiviral vector infected ducks post-infection with APEC. The samples of NLRP3-lentiviral vector infected and NC-lentiviral vector-infected ducks were collected at 1, 2, and 3 dpi. And all these mRNA fold changes were calculated using the NLRP3-lentiviral vector-infected ducks vs. NC-lentiviral vector infected ducks at the same time point. The fold change was calculated by the 2^−Δ*Ct*^ method and expressed as the means ± SD (*n* = 3), and the student's *t* test was performed to evaluate the differences. *Significant difference (*P* < 0.05); **highly significant difference (*P* < 0.01); dpi, days post-infection.

We also detected the mRNA expression levels of inflammatory cytokines IL-1β, IL-18, and TNF-α in the liver, spleen, and brain after the *E. coli* infection. As shown in Figures 4A–C, the expression of IL-1β displayed significant up-regulation in the liver, spleen, and brain at 2 and 3 dpi. IL-18 and TNF-α displayed up-regulation in these three tissues at all indicated times (Figures 4D–I), but in general, the fold changes in the brain are less than those in the liver and spleen (Figures 4D–I). The results show that injection with the NLRP3-lentiviral vector *in vivo* could increase the duNLRP3 mRNA expression level in most of the tissues with lower duNLRP3 background expression, induce the production of inflammatory factors, and a reduction in the bacterial growth was observed. The results indicate that duNLRP3 effectively participates in the innate immune response to *E. coli in vivo*.

### DuNLRP3 is critical for the innate immune response against *E. coli* infection *in vitro*

**Figure 5:**
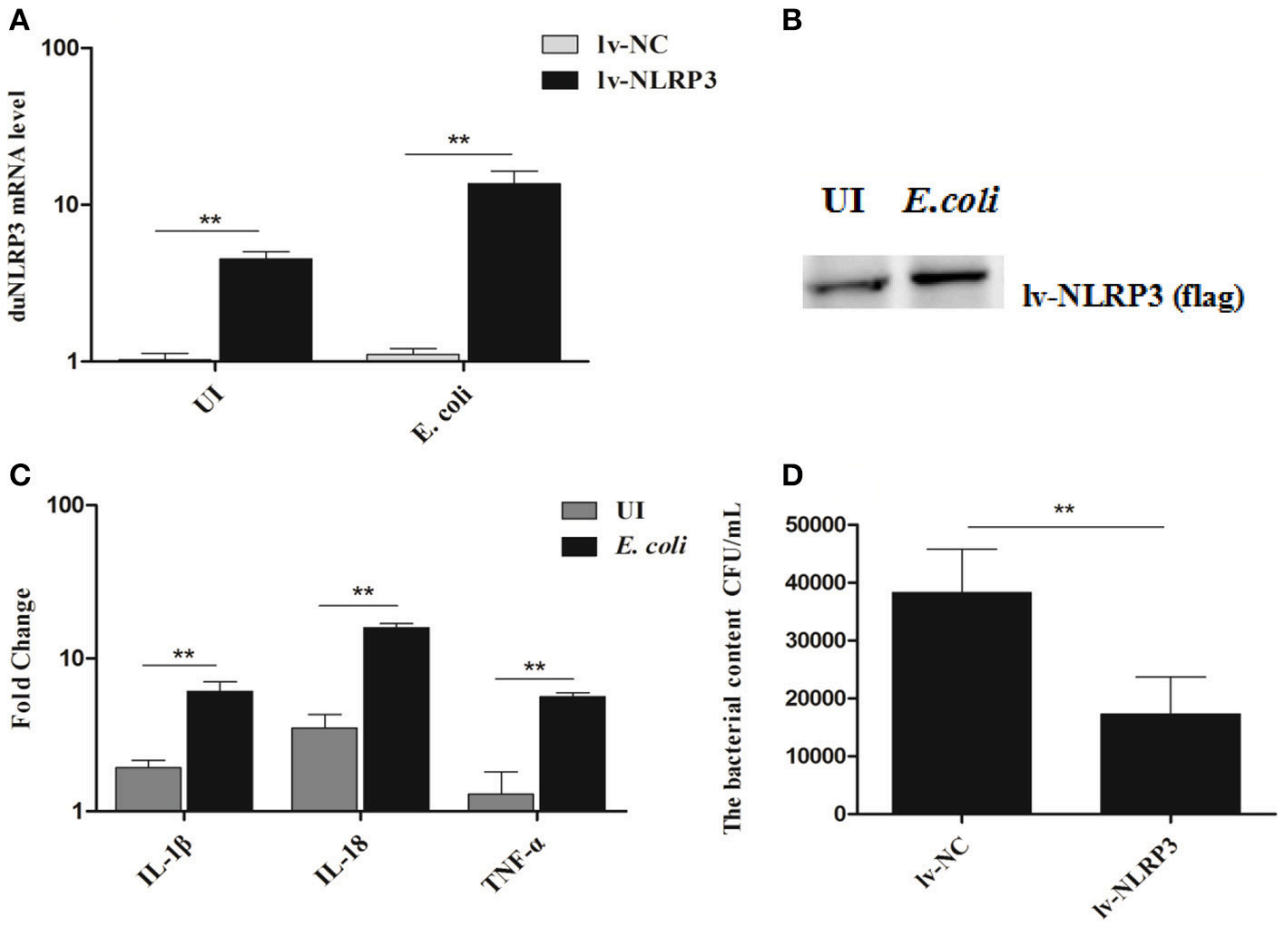
Overexpression of duNLRP3 increases inflammatory cytokine production and reduces bacterial content in DEFs. DEFs were seeded in 6-well plates and infected with 20 μL of 10^8^ TU/mL NLRP3-lentiviral vector (MOI = 10) for 48 h. The cells were then infected or uninfected (UI) with 1 × 10^4^ CFU/mL *E.coli* for 3 h and harvested at 4 hpi for **(A)** duNLRP3 mRNA expression level, the *E.coli*-infected or UI DEFs were used as controls respectively, **(B)** duNLRP3 protein expression level, **(C)** inflammatory cytokine mRNA expression level, the corresponding NC-lentiviral vector infected DEFs as the control, and **(D)** intracellular bacterial CFU detection. The data in **(A)** and **(C)** were analyzed by the 2^−ΔΔ*Ct*^ method. All data were expressed as the means ± SD (*n* = 3), and the student's *t* test was performed to evaluate the differences. **Highly significant difference (*P* < 0.01).

To further verify the antibacterial effect of duNLRP3 on *E. coli* infection, a series of experiments *in vitro* were performed. Firstly, the duNLRP3 was successfully overexpressed in DEFs via the NLRP3-lentiviral vector infection. DuNLRP3 showed higher up-regulation in both mRNA and protein expression level, especially after the *E. coli* infection (Figures 5A,B, *P* < 0.01). In addition, the mRNA expression levels of IL-1β, IL-18, and TNF-α induced by *E. coli* infection were significantly up-regulated in comparison with the NC-lentiviral vector group (Figure 5C, *P* < 0.01). To explore the antibacterial ability of duNLRP3 against *E. coli*, DEFs were infected with *E. coli* after the NC- and NLRP3-lentiviral vector infection, and then the *E. coli* counts were calculated as shown in Figure 5D. The number of *E. coli* was significantly lower in NLRP3-lentiviral vector-infected DEFs (*P* < 0.01).

**Figure 6:**
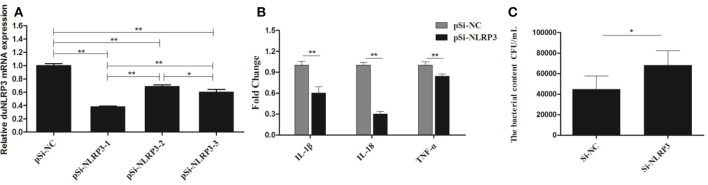
DuNLRP3 knockdown decreases antibacterial activity and inflammatory cytokine production in DEFs. DEFs were seeded in 6-well plates and transfected with 1.0 μg/well of pSi-RNA. **(A)** At 36 hpt, the cells were collected for duNLRP3 detection. **(B)** At 36 hpt, the DEFs were infected with 1 × 10^4^ CFU/mL APEC O1K1 for 3 h and harvested at 4 hpi for inflammatory cytokine detection. **(C)** The cell samples collected for **(B)** were also lysed with 10-fold serial dilution and plated onto eosin methylene blue nutrient agar to calculate intracellular bacterial CFU. The data in **(A)** and **(B)** were analyzed by the 2^−ΔΔ*Ct*^ method. All data were expressed as the means ± SD (*n* = 3), the ANOVA test **(A)** and the student's *t* test **(B,C)** were performed to evaluate the differences. *Significant difference (*P* < 0.05); **highly significant difference (*P* < 0.01).

Correspondingly, we transfected the DEFs with pSi-NLRP3s or pSi-NC for 36 h, as shown in Figure 6A, PSi-NLRP3-1, pSi-NLRP3-2, and pSi-NLRP3-3 were all able to decrease the mRNA expression of duNLRP3 in DEFs, but the pSi-NLRP3-1 had the strongest interference ability, so we chose it for further study (*P* < 0.01). After 36 hpt, the cells were infected with 10^4^ CFU/mL of *E. coli*, and then the cell samples were collected for detection of the cytokines and bacterial count. As shown in Figure 6B, the knockdown of duNLRP3 significantly impaired the mRNA expression levels of IL-1β, IL-18, and TNF-α induced by *E. coli* infection (*P* < 0.01). In addition, the *E. coli* content was obviously higher in the cells transfected with pSi-NLRP3 than the pSi-NC-transfected control cells (Figure 6C, *P* < 0.05). These results verify that duNLRP3 could induce the production of inflammatory factors and inhibit the bacterial growth *in vitro*. Therefore, duNLRP3 is critical for the innate immune response to *E. coli* infection.

## Discussion

Although the expression profile and tissue distribution of NLRP3 has been determined in mice and chickens (22, 23), this information has not yet been made available for ducks. In the present study, the full-length CDs of duNLRP3 were firstly obtained and predicted by the SMART tool. We found that the duNLRP3 contained PYD, NACHT, and six LRR motifs (Figures 1A,B). All these motifs are in line with the NLRs (5). Evolutionary analysis showed that the duNLRP3 has a close genetic relationship to the NLRP3 of *Anas platyrhyncho* (Figure 1C).

Previous studies have shown that NLRP3 could be expressed in immune cells, granulocytes, chondrocytes, and non-keratinizing epithelial cells in humans (24, 25). Ye et al. showed that the lung and trachea have a higher level of chicken-NLRP3 expression than other tissues (23). In BALB/c mice, the NLRP3 expression was higher in the inguinal lymph nodes than other tissues (22). These reports indicate that NLRP3 has a different tissue distribution in different species. In the present study, higher expression of duNLRP3 was found in the pancreas, heart, gizzard, and cerebellum (Figure 2A). These results proved that NLRP3 is widely and differently distributed in healthy duck tissues. Studies have pointed out that lipopolysaccharide stimulation could highly induce NLRP3 mRNA expression in bone marrow-derived macrophages from mice (26). In our study, the duNLRP3 mRNA expression in the liver (the main target organ of *E. coli* infection) was kept elevated and reached a peak at 3 dpi. But in the spleen, it exhibited down-regulation from 1 to 3 dpi, and in the brain, the change was not obvious until 3 dpi (Figures 2B–D). All these results suggest that duNLRP3 could participate in the immune process caused by *E. coli*. Such information could also suggest site-specific functions of duNLRP3 in inflammatory responses.

As one of the most characterized innate immune receptors, NLRP3 could recognize a wide range of microbial or danger signals and mediate the immune response (27). For example, NLRP3 plays an important role in viral infections, such as hepatitis C, influenza, and the modified vaccine virus Ankara (28–30). In addition, some bacteria could also induce the activity of NLRP3, such as the *Listeria monocytogenes, Streptococcus pyogenes*, and *E. coli* (31–33). To confirm that duNLRP3 has a certain anti-*E. coli* function, we detected the bacterial content in both duNLRP3-overexpressed tissues and DEFs. The results showed significant reductions in the bacterial content in the liver and spleen from the NLRP3-lentiviral vector-injected group (Figures 3C,D). Infection of the NLRP3-lentiviral vector in DEFs also significantly inhibited bacteria growth (Figure 5D, *P* < 0.01). In accordance with these results, the bacteria content was significantly higher when the DEFs were transfected with pSi-NLRP3 (Figure 6C, *P* < 0.05). Similarly, previous studies have pointed out that mice lacking NLRP3 were more susceptible to group B *Streptococcus* and *Citrobacter rodentium* infection when compared with wild-type mice (34, 35). NLRP3 combined with ASC and caspase-1 is critical for host defense against *Burkholderia pseudomallei* infection in the lungs (36). Our results clearly demonstrate that duNLRP3 has an antibacterial function in *E. coli* infection.

IL-1β and IL-18 are the major inflammatory cytokines induced by the NLRP3, and their expression profile has been fully studied for a variety of microbial infections. For example, studies have pointed out that *Staphylococcus aureus, Salmonella*, Sendai virus, and influenza virus could induce IL-1β secretion in NLRP3 inflammasome activation (31, 37, 38). IL-1β is an important inflammatory cytokine that could be regulated by a multimeric protein complex in host cells and secreted into the extracellular environment. The inflammatory response is then amplified by paracrine or autocrine mechanisms (39). Our previous study has showed that IL-1β, IL-6, and IL-8 in duck and DEFs increase somewhat after *E. coli* infection (40). In this study, we demonstrated the role of duNLRP3 in the expression of inflammatory cytokines IL-1β, IL-18, and TNF-α mRNA after *E. coli* infection. The results show that IL-1β, IL-18, and TNF-α in the liver, spleen, and brain of the NLRP3-lentiviral vector-injected group mostly increased after *E. coli* infection (Figures 4). The same results were also found in the NLRP3-lentiviral vector-infected DEFs, especially after *E. coli* infection (Figure 5C). The knockdown of duNLRP3 in DEFs suggested that duNLRP3 could reduce the production of inflammatory cytokines (Figure 6B). All these results suggest that duNLRP3 could induce the mRNA expression of IL-1β, IL-18, and TNF-α during *E. coli* infection, shows that duNLRP3 plays an important regulatory role in the innate immune response to *E. coli*.

NLRP3 is one of the most characterized innate immune receptors and has been fully studied in many species, but not in waterfowl. Our research could help to understand the interaction between PRRs and pathogen-associated molecular patterns, as well as their signal transduction pathways. Based on the crucial role of NLRP3 in antibacterial innate immunity, we cloned and characterized duNLRP3, predicted its main functional domains, detected its tissue distribution, and studied its antibacterial function in ducks and DEFs. This study could lay the foundation for understanding NLRP3's antibacterial innate immunity mechanism.

## Author contributions

RL wrote the manuscript and performed the most of the experiments. JL collected the samples and extracted the sample RNA. XH and SH kept animals. HW and TX analyzed the data with support from NL. TC reviewed and polished the article. LW designed the study.

### Conflict of interest statement

The authors declare that the research was conducted in the absence of any commercial or financial relationships that could be construed as a potential conflict of interest.
